# Effect of Spatial Inhomogeneities on the Membrane Surface on Receptor Dimerization and Signal Initiation

**DOI:** 10.3389/fcell.2016.00081

**Published:** 2016-08-12

**Authors:** Romica Kerketta, Ádám M. Halász, Mara P. Steinkamp, Bridget S. Wilson, Jeremy S. Edwards

**Affiliations:** ^1^Department of Pathology, University of New Mexico Health Sciences CenterAlbuquerque, NM, USA; ^2^Department of Mathematics and Mary Babb Randolph Cancer Center, West Virginia UniversityMorgantown, WV, USA; ^3^Cancer Center, University of New Mexico Health Sciences CenterAlbuquerque, NM, USA; ^4^Department of Chemical and Biological Engineering, University of New MexicoAlbuquerque, NM, USA; ^5^Department of Chemistry and Chemical Biology, University of New MexicoAlbuquerque, NM, USA; ^6^Department of Molecular Genetics and Microbiology, University of New MexicoAlbuquerque, NM, USA

**Keywords:** spatial stochastic modeling, membrane domains, ErbB receptors, ErbB2, ErbB3

## Abstract

Important signal transduction pathways originate on the plasma membrane, where microdomains may transiently entrap diffusing receptors. This results in a non-random distribution of receptors even in the resting state, which can be visualized as “clusters” by high resolution imaging methods. Here, we explore how spatial in-homogeneities in the plasma membrane might influence the dimerization and phosphorylation status of ErbB2 and ErbB3, two receptor tyrosine kinases that preferentially heterodimerize and are often co-expressed in cancer. This theoretical study is based upon spatial stochastic simulations of the two-dimensional membrane landscape, where variables include differential distributions and overlap of transient confinement zones (“domains”) for the two receptor species. The *in silico* model is parameterized and validated using data from single particle tracking experiments. We report key differences in signaling output based on the degree of overlap between domains and the relative retention of receptors in such domains, expressed as escape probability. Results predict that a high overlap of domains, which favors transient co-confinement of both receptor species, will enhance the rate of hetero-interactions. Where domains do not overlap, simulations confirm expectations that homo-interactions are favored. Since ErbB3 is uniquely dependent on ErbB2 interactions for activation of its catalytic activity, variations in domain overlap or escape probability markedly alter the predicted patterns and time course of ErbB3 and ErbB2 phosphorylation. Taken together, these results implicate membrane domain organization as an important modulator of signal initiation, motivating the design of novel experimental approaches to measure these important parameters across a wider range of receptor systems.

## Introduction

The plasma membrane is the initiation site for signaling pathways that govern cell differentiation, proliferation and survival (Groves and Kuriyan, 2010; Radhakrishnan et al., 2012). The membrane provides a platform for the reversible binding of ligands to receptors, initiating critical processes such as dimerization, activation of catalytic activity and recruitment of binding partners (Groves and Kuriyan, 2010). Given its importance in cell signaling, the structure and composition of membranes have been probed by many different groups. Singer and Nicholson, in their landmark paper of the fluid mosaic model, proposed membranes to be largely homogenous with randomly distributed mixtures of integral membrane proteins and lipids (Singer and Nicolson, 1972). However, the authors also showed electron microscopy images of major histocompatibility antigen “patches,” providing early evidence for membrane organization. Since then, considerable evidence has accumulated showing that membrane proteins and lipids can be transiently confined in specific domains (Kaizuka et al., 2007; Chung et al., 2010; Treanor et al., 2010; Radhakrishnan et al., 2012; Goñi, 2014). The anomalous diffusion of membrane constituents, observed through single molecule tracking methods (Fujiwara et al., 2002), is likely due, at least in part, to their transient entrapments within heterogeneous domains (Marguet et al., 2006). Multiple theories exist to explain the richness of the plasma membrane topography, including lipid rafts which are enriched in unsaturated fatty acids and cholesterol (Pike, 2003), corrals formed by the actin cortical cytoskeleton network (Jaqaman et al., 2011; Kalay, 2012; Cambi and Lidke, 2015) and protein islands (Lillemeier et al., 2006). Even very short periods of confinement within domains give rise to lateral heterogeneity and an uneven distribution of proteins on the membrane surface that can be captured in “snap-shot” images by electron microscopy of membrane rip-flips (Wilson et al., 2000; Prior et al., 2001; Andrews et al., 2009). More recently, super-resolution microscopy methods have also been employed to document the clustering of membrane proteins (van den Dries et al., 2013; Itano et al., 2014). The exchange of proteins between domains is highly variable, ranging from very low exchange rates observed in yeast membranes (Spira et al., 2012) to very rapid exchanges described for the EGFR in mammalian cell membranes (Low-Nam et al., 2011).

Many important receptors exhibit varying degrees of clustering prior to ligand engagement, including members of the EGFR/ErbB family (Nagy et al., 2002; Yang et al., 2007) and the ITAM-bearing immunoreceptors (FcεRI, BCR, TCR) (Pike, 2003; Lillemeier et al., 2006; Andrews et al., 2009; Tolar et al., 2009; Treanor et al., 2010; Dinic et al., 2015). Experimental evidence has suggested that membrane domains can both enhance and inhibit signaling in different settings (Marmor and Julius, 2001; Miura et al., 2001; Douglass and Vale, 2005; Allen et al., 2007; Bénéteau et al., 2008; Ganguly et al., 2008). Computational studies have also supported the concept that membrane organization has cell and receptor-specific outcomes (Lim and Yin, 2005; Hsieh et al., 2008; Costa et al., 2011; Abel et al., 2012; Kalay et al., 2012). For example, the formation of different signaling clusters has been proposed to support distinct TCR signaling patterns (Singleton et al., 2009). Vale and colleagues recently demonstrated in model membranes that phase separation of signaling partners can create distinct signaling compartments (Su et al., 2016). Members of the ErbB family of receptor tyrosine kinases have been shown to have distinct distribution patterns on cancer cell membranes (Yang et al., 2007; Steinkamp et al., 2014), leading to computational studies from our group that predict the impact of critical variables such as receptor co-expression, density and dimer off-rates (Hsieh et al., 2008; Pryor et al., 2013, 2015).

Deterministic models based upon Ordinary Differential Equations (ODEs) are not well suited to explore spatial aspects of signaling, since they assume molecules in a system are well mixed. Stochastic modeling approaches offer greater flexibility to consider effects of membrane topography, receptor clustering and diffusion dynamics on signaling events (Mayawala et al., 2006; Nicolau et al., 2006; Hsieh et al., 2008; Costa et al., 2009; Chaudhuri et al., 2011). These versatile mathematical models provide a platform for rapid exploration of key factors that are difficult to vary (and measure) experimentally. In this study, we take advantage of this powerful approach to consider the effect of two parameters, membrane domain overlap and domain retention, on ErbB3 and ErbB2 homo- and heterodimerization. Our group previously evaluated the domain occupancy and distribution of ErbB2 and ErbB3 stably expressed as recombinant proteins in Chinese Hamster Ovary (CHO) cells (Steinkamp et al., 2014; Pryor et al., 2015). Analysis of dual-color single particle tracking data, which permitted independent observations of each species, indicated that domains confining the two ErbB receptors were only partially overlapping in the CHO cell membrane (Pryor et al., 2015). We then built a spatial stochastic model based upon this distribution, as well as experimentally measured values for dimer off-rates, kinase/phosphatase activity and receptor diffusion (Pryor et al., 2015). However, we speculate that the degree to which there is differential segregation of these two closely related receptors will vary widely as a property of cell type, because of dissimilar receptor ratios, density, cytoskeletal features, membrane composition and on-going signal transduction from other cell surface receptors triggered by circulating or local ligands. In this paper, we focus on two specific parameters that affect the degree to which ErbB2 and ErbB3 experience periods of co-confinement: domain overlap and retention, where the latter is expressed as a function of escape probability.

## Materials and methods

### Spatial stochastic model for ErbB2 and ErbB3 homo- and hetero-dimerization

#### Reactions

The spatial stochastic model of ErbB2 and ErbB3 interactions was described previously (Pryor et al., 2015). Briefly, the model includes two members of the EGFR family, ErbB2 and ErbB3, which diffuse within the simulation space and interact with each other.

The following reactions are accounted for in the model:

- Dimerization: Homo- and heterodimerization of ErbB2 and ErbB3 receptors.
- Phosphorylation: Receptors are phosphorylated through intrinsic phosphorylation rates.
- Dephosphorylation: Receptors are dephosphorylated through experimentally determined dephosphorylation rates.
- Dissociation: Dimer dissociation occurs through experimentally determined dimer off rates.

We assume that the dimerization of receptors occurs through the interaction of the dimerization arms on the extracellular domain of receptors. In the absence of ligand, the ErbB3 extracellular domain fluxes from a closed (tethered) to an open (dimer-competent) conformation. The open conformation of ErbB3 is stabilized by ligand binding (Pryor et al., 2015). Unliganded ErbB3 is assumed to be predominately closed (99.99% closed). At any given time step, there is a 10^−4^ probability for unoccupied ErbB3 receptors to assume the upright dimer-competent state while all ligand-bound ErbB3 monomers are dimer-competent (Hsieh et al., 2008). ErbB3 ligand concentrations vary in the simulations as described in the legends. ErbB2 receptors are assumed to be in open conformation and dimerization competent (Cho et al., 2003; Garrett et al., 2003). In the model, ErbB2 has a single representative tyrosine phosphorylation site based on uniform dephosphorylation kinetics over two tested phosphorylation sites (Pryor et al., 2015). ErbB3 has two representative phosphorylation sites based upon (Y1289; Y1197). Table 1 lists the reaction parameters used in our model including receptor dimerization, phosphorylation/dephosphorylation, and receptor dissociation as previously described (Pryor et al., 2015). For receptor phosphorylation events, the model takes into consideration the asymmetric orientation of kinase domains which occurs during ErbB receptor activation (Ward and Leahy, 2015). Reactions are governed by binding radii estimated using SMOLDYN, a software application that takes into consideration receptor on-rates, diffusion coefficients and simulation time steps to construct a binding radius (Andrews and Bray, 2004). An unbinding radius of 5 times the binding radius was used to decrease rebinding events.

**Table 1:** **Model parameters of receptor monomers and dimers**.

	**ErbB2**	**ErbB3**	**ErbB2**	**ErbB3**	**ErbB2**
			**ErbB3**	**ErbB3**	**ErbB2**
Diffusion coefficient (μm^2^/s)^a^^,^^b^	0.0272	0.013	0.015	0.0185	0.015
Diffusion coefficient (phosphorylated) (μm^2^/s)^a^^,^^b^			0.0046	0.0028	0.015
Dimer on rate (μm^3^/s)^c^			0.00009	0.00009	0.00009
Dimer off rate (0 ligand) (1/s)^a^^,^^b^			0.436	0.436	4.36
Dimer off rate (1 ligand) (1/s)^b^			0.408	0.234	
Dimer off rate (2 ligand) (1/s)^b^				0.13	
Basal Phosphorylation rate (1/s)^d^^,^^e^	0.073	0.00007			
Phosphorylation rate (1/s)^d^^,^^e^	0.146	0.078			
Dephosphorylation rate (1/s)^a^	0.2	0.013			
		(PY1197)			
		0.06			
		(PY1289)			

a Pryor et al. (2015).

b Steinkamp et al. (2014).

c Pryor et al. (2013).

d Kleiman et al. (2011).

e Shi et al. (2010).

#### Simulation landscape

**Figure 1:**
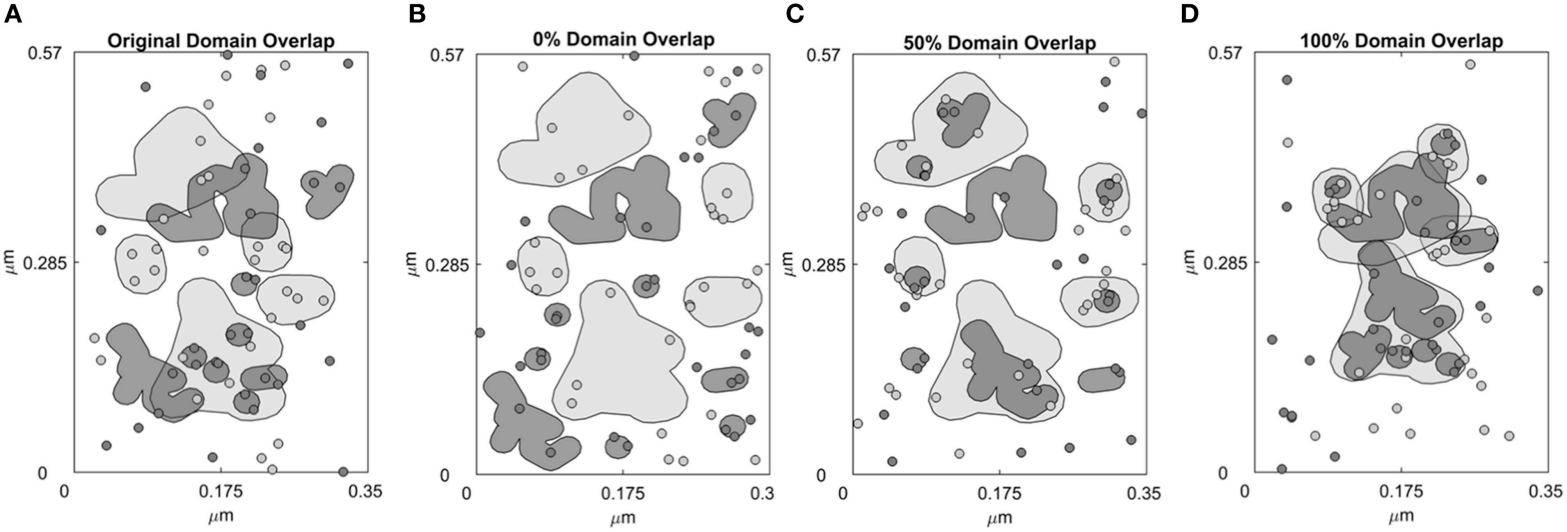
**Four domain configurations of the simulation space**. Simulation space was partitioned into receptor-specific domains with defined domain overlaps. **(A)** A simulation space that mimics the domain properties of CHO cells overexpressing ErbB2 and ErbB3 based on domain analysis of SPT data. ErbB2 (light gray, shaded) and ErbB3 (dark gray, shaded) membrane domains overlap by 42.4%. ErbB2 receptors (light gray, circled) and ErbB3 receptors (dark gray, circled) are randomly distributed within their own domains as well as outside the domains (white region). **(B–D)** Domains were rearranged to create a simulation space where the ErbB2 and ErbB3 domains are completely non-overlapping (0% overlap, **B**), partially overlapping (50% overlap, **C**) or completely overlapping (100% overlap, **D**). In the initial configuration, ErbB2 and ErbB3 receptors were positioned to randomly occupy their respective domains.

The simulation landscape contains receptor specific domains (Figure 1A) and receptors can diffuse across domains and domain-free areas. An exit penalty limits receptor escape from the domains. Figure 1A depicts domains that were identified in previous work (Pryor et al., 2015). Represented by a rectangular box measuring 0.1995 μm^2^ in area (Figure 1A), the space contains 5 ErbB2 and 9 ErbB3 receptor domains. These domains were derived from domain analysis of two-color single particle tracking data where ErbB3 was labeled with HRG-conjugated quantum dot (QD) and HA-tagged ErbB2 was labeled with anti-HA Fab conjugated QD (Pryor et al., 2015). The total ErbB2 domain area is 0.0502 μm^2^; the total ErbB3 domain area is 0.0274 μm^2^.The free area outside the domains is 0.1219 μm^2^. We then created three distinct domain overlap conditions for comparison:

- 100% overlap: 100% of the ErbB3 domain area is overlapping with the ErbB2 domain area. This resulted in complete mixing of ErbB3 and ErbB2 domains (Figure 1D).
- 50% overlap: 50% of the ErbB3 domain area is overlapping with ErbB2 domain area. This resulted in partial overlapping of ErbB3 and ErbB2 domains (Figure 1C).
- 0% overlap: 0% of the ErbB3 domain area is overlapping with the ErbB2 domain area. This resulted in complete separation of ErbB3 and ErbB2 domains (Figure 1B).

#### Number and density of receptors

The model was populated with 50,000 ErbB2 and 50,000 ErbB3 receptors/cell. Since the total area of a cell is 314.16 μm^2^ (with a diameter of 10 μm), this translates into a receptor *density* of ~159 receptors/μm^2^ for each receptor. Adjusted for a simulation area of 0.1995 μm^2^, the total *number* of receptors is 31 of each receptor species.

#### Receptor diffusion

Receptor diffusion occurs in the two dimensional membrane simulation space (x and y direction) through Brownian motion. Receptor jumps in these two directions are calculated using diffusion coefficients generated from SPT data and normally distributed random numbers.

#### Boundary conditions

As in Pryor et al. (2015) and Pryor et al. (2013), the periodic boundary condition is applied to the edges of the simulation space. If a receptor jump takes the receptor across the edge of the simulation space, the jump distance is divided between the distances covered before and after the boundary is crossed. The receptor then traverses the distance to the boundary and the remaining distance is calculated from the opposite edge of the simulation space. Hence, the receptor “re-enters” the simulation space from the opposite boundary. Reflective boundary conditions are applied when a receptor reaches the edge of a membrane domain. Like the periodic boundary conditions, the jump distance is divided between the distances covered before and after reaching the boundary. A probability for crossing/escaping from the membrane boundary is calculated and if the probability of escaping is not met, then the receptor hits the boundary and is deflected back into the domain. If the probability of escape is met, then the receptor continues across the boundary. Escape rates in Pryor et al. (2015) were estimated by parameter fitting to the ratio of domain-confined receptors experimentally measured in CHO cell membranes; this rate is a key variable of the present study (Table 2).

**Table 2:** **Escape rates of receptor monomers and dimers**.

	**ErbB2**	**ErbB3**	**ErbB2**	**ErbB3**	**ErbB2**
			**ErbB3**	**ErbB3**	**ErbB2**
Nominal escape rate^a^	0.5128	0.2401	0.3764	0.2401	0.5128
Escape rate reduced by 1/2^b^	0.2564	0.1200	0.1882	0.1200	0.2564
Escape rate reduced by 1/4^b^	0.1282	0.0600	0.0941	0.0600	0.1282

a Pryor et al. (2015).

b Simulation data in this paper.

#### Simulation code

Input files containing the initial simulation space, receptor locations and ligand concentrations are generated in Matlab. These files are then accessed by a program written in Fortran, which simulates brownian diffusion and molecular interactions between the two receptors. At the end of the simulations, all output files are processed in Matlab for analysis of results. Code is available upon request.

## Results

### Domain overlap affects the frequency of hetero-interactions and receptor phosphorylation events

It is unknown to what extent different receptors share the same membrane domains, how fluid these domains are over time, and whether activation of receptors alter domain overlap. Therefore, we explored these possibilities through simulations, reporting results as changes in homo- and hetero-dimerization and phosphorylation status. Unlike prior work fit to cells overexpressing ErbB family members (Pryor et al., 2013, 2015), we used receptor densities within the range of expression values expected for normal cells (50,000 receptors/cell). The simulation landscape included either no domains or ErbB2 and ErbB3-specific domains with partial, full or no overlap (Figure 1).

**Figure 2:**
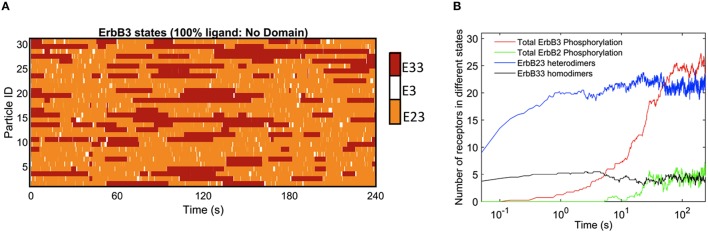
**Kinetics of ErbB3 dimerization and phosphorylation**. **(A)** Representative plot of individual ErbB3 receptors showing changes in receptor state over time. ErbB3 receptors cycle between homodimer, heterodimer and monomer states. **(B)** Plot showing the kinetics of dimer formation and phosphorylation of ErbB2 and ErbB3. ErbB2/3 heterodimer and ErbB3/3 homodimer formation are plotted with total ErbB2 and ErbB3phosphorylation over time for 100% ligand in the absence of domains. Data in B are the averages of 4 runs.

The rapid cycling of ErbB3 receptors through different states is illustrated in Figure 2, where simulations were initially performed in a landscape lacking domains. Here, ligand-bound ErbB3 freely diffuse, encountering other ErbB3 or ErbB2 monomers with no barriers imposed. They constantly cycle through homodimer (red), heterodimer (orange) and monomer (white) states by binding and unbinding to other receptors as they diffuse through the simulation space (Figure 2A). Off-rates for hetero- and homodimers are assigned probabilities based upon experimental measures for unoccupied and ligand bound dimers (Steinkamp et al., 2014). The catalytic activity of each monomer in a dimer is tracked throughout the simulation. Activity is dependent on the stochastically-governed orientation of the monomer in the asymmetric model, where one of the monomers is the “activator” and the other monomer is the “receiver.” Further, ErbB3 monomers are assumed to require phosphorylation by a “receiver” ErbB2 in a prior hetero-dimerization event. A phosphorylated ErbB3 monomer remains a competent “receiver” during subsequent encounters only until it is dephosphorylated. Simulation time steps are 1 × 10^−6^ s and observations are recorded every 0.05 s. Plots in Figure 2B show that dimerization is already occurring by the earliest observation interval and continues to rise over the first 10 s of the simulation. Phosphorylation kinetics are delayed, observable within 0.5 s of the simulation and rising to steady state values by 50 s.

**Figure 3:**
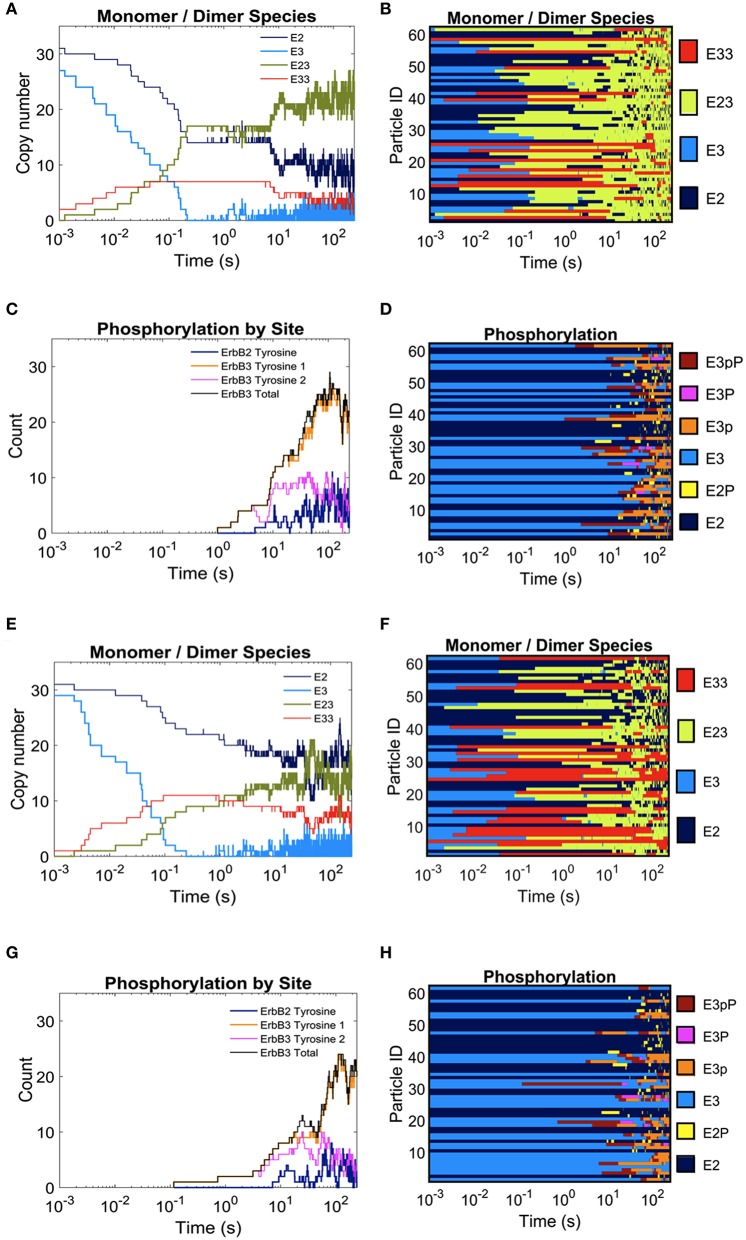
**The effect of overlapping domains on ErbB2/ErbB3 dimerization and phosphorylation kinetics with 100% ligand-bound ErbB3**. Plots for the completely overlapping domain configuration **(A–D)**: The kinetics of dimer formation **(A)**, representative plots of dimerization state for individual receptors over the simulation time **(B)**, the kinetics of receptor phosphorylation **(C)**, and a representative plot of phosphorylation state for receptors over time **(D)**. **(E–H)**: Plots for the non-overlapping domain configuration. Plots are arrayed as in **(A–D)**.

In Figure 3, we report the effect of adding domains to these simulations. The extreme cases of completely overlapping vs. non-overlapping ErbB2 and ErbB3 domains are shown in Figures 3A–H. Color keys in these plots indicate shifting profiles of monomers and dimers, as well as report phosphorylation states. Clearly, confinement in shared domains favors heterodimer interactions with a corresponding decrease in ErbB3 homodimers and ErbB2 monomers (Figures 3A,B). Phosphorylation kinetics is affected by co-confinement with a delayed but steep rise in phosphorylation (Figures 3C,D). Therefore, the overall signaling response is likely increased with shared domains.

**Figure 4:**
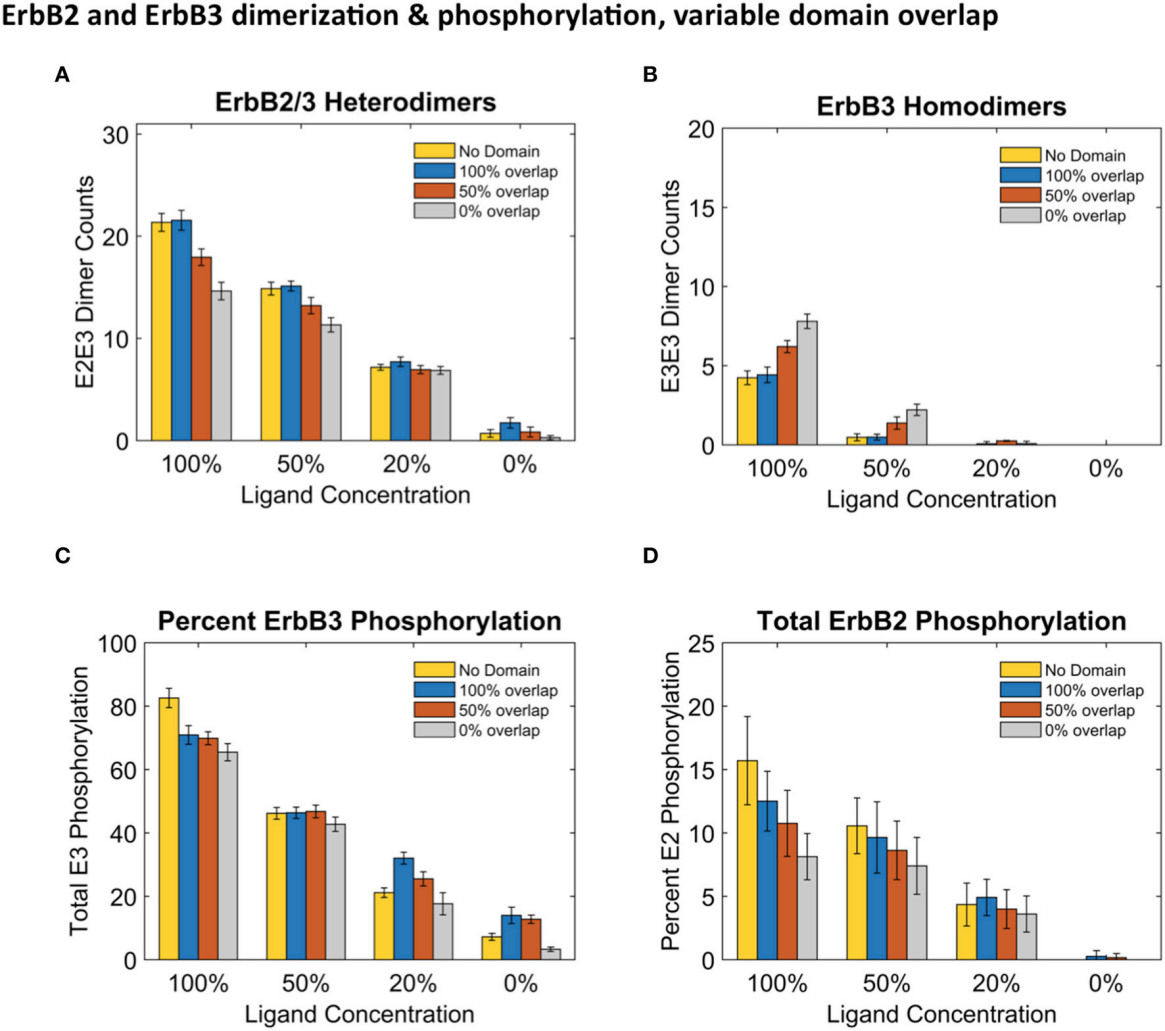
**Overlapping domains influence dimer formation and phosphorylation. (A,B):** Dimer counts across different ligand concentrations with 4 different membrane configurations- 100% (blue bars), 50% (orange bars), and 0% overlap (gray bars) as well as no domain simulations (yellow bars) for ErbB2/ErbB3 heterodimers **(A)** and ErbB3 homodimers **(B)**. **(C,D):** Total receptor phosphorylation across different ligand concentrations and all four domain configurations for ErbB3 **(C)** And ErbB2 **(D)**. All bars are the averages of 4 runs ± standard deviation.

Results in Figure 4 report dimers at steady state (240 s) using the three distinct domain configurations shown in Figures 1B–D as well as no domain configuration. Simulations with completely overlapping domains produced the greatest number of heterodimers regardless of ligand concentration, although the greatest difference can be seen with 100% ligand (Figure 4A). At lower ligand concentrations, the effect of overlapping domains on dimer formations was diminished. This phenomenon is best explained by segregation of the few ligand bound receptors. ErbB3 homodimers displayed the opposite trend to that of heterodimers, where the highest number of homodimers were seen when ErbB3 domains did not overlap with ErbB2 (Figure 4B). This was notable for conditions of 100% and 50% liganded ErbB3.

Steady state phosphorylation levels are also affected by the configuration of domains (Figures 4C,D). Phosphorylation levels of both ErbB2 and ErbB3 decreased as domain overlap decreased, highlighting the importance of heterointeractions for maximal signaling. ErbB2 phosphorylation was most affected by domain overlap, particularly in simulations with 100% liganded ErbB3 (Figure 4D). Note that ErbB3 phosphorylation, which is heavily dependent on interactions with ErbB2 is not favored under conditions where ErbB2 homodimers are predominant.

### Stronger domain retention affects receptor dimerization and phosphorylation events only when the domains partially overlap or non-overlap

**Figure 5:**
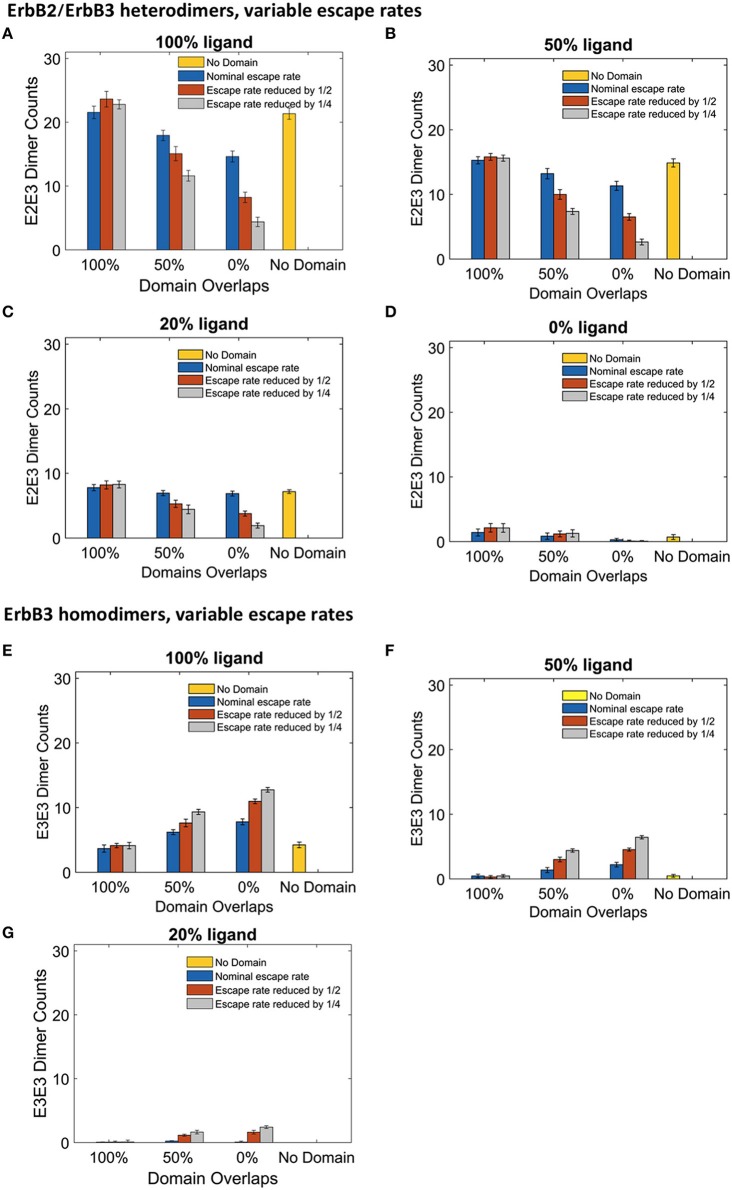
**The effect of changes in domain retention on ErbB2/3 heterodimer and ErbB3/3 homodimer counts across different ligand concentration and domains**. Dimer counts across different membrane configurations, ligand concentration and three different escape rates- nominal escape rate (blue bars), escape rate reduced by ½ (orange bars), and escape rate reduced by ¼ (gray bars) as well as no domain simulations (yellow bars). **(A)** ErbB2/3 heterodimer for 100% liganded ErbB3. **(B)** ErbB2/3 heterodimer for 50% liganded ErbB3. **(C)** ErbB2/3 heterodimer for 20% liganded ErbB3. **(D)** ErbB2/3 heterodimer for 0% liganded ErbB3. **(E)** ErbB3/3 homodimer for 100% liganded ErbB3. **(F)** ErbB3/3 homodimer for 50% liganded ErbB3. **(G)** ErbB3/3 homodimer for 20% liganded ErbB3. The ErbB3/3 homodimer count was 0 for 0% liganded ErbB3. All bars are the averages of 4 runs ± standard deviation.

Although the clustering of receptors in domains is important for signaling, little is known about the movement of receptors into and out of membrane domains or the extent to which this movement is altered with receptor activation. Since it is difficult to measure experimentally receptor residency times within domains, Pryor et al estimated an escape rate based on the ratio of domain-confined to free receptors in CHO cells under low ligand conditions (Pryor et al., 2015). To examine the effect of this parameter on signaling outcome, we ran simulations where we varied the escape rate to model changes in domain retention. The affinity of receptors for their domains was increased by reducing the escape rate of both monomers and dimers. We compared simulations run with the original nominal escape rate, or with the escape rate reduced by ½ or ¼. The effect of these escape rates were examined with different ligand concentrations in the four domain overlap configurations (Figure 5). Reducing the escape rates had no effect on heterodimer formation for domains that were completely overlapping. However, when the domains were partially overlapping or non-overlapping, heterodimer formation was significantly reduced as the escape rate decreased. For instance, in the case of 100% liganded ErbB3, when the escape rate was reduced to ¼ and the domains were partially overlapping, the number of heterodimers at steady state was 35% lower than with the original escape rate. With non-overlapping domains, heterodimers were reduced by 70% (Figure 5A). Similar trends were seen in 50% and 20% ligand conditions (Figures 5B,C). With unliganded ErbB3, heterodimerization was rare (Figure 5D). With completely overlapping domains, reducing the escape rates did not affect erbB3 homodimer formation either (Figures 5A–D). With overlapping domains, reducing the escape rate increased ErbB3 homodimers for partially and non-overlapping domains (Figures 5E–G). Escape rates ¼ of the original rate yielded maximum increase of 63%, which occurred with non-overlapping domains and 100% ligand (Figure 5E). Similar trends were seen with lower ligand concentrations (Figures 5F,G). Unliganded ErbB3 is not shown since there were no homodimers in this condition.

**Figure 6:**
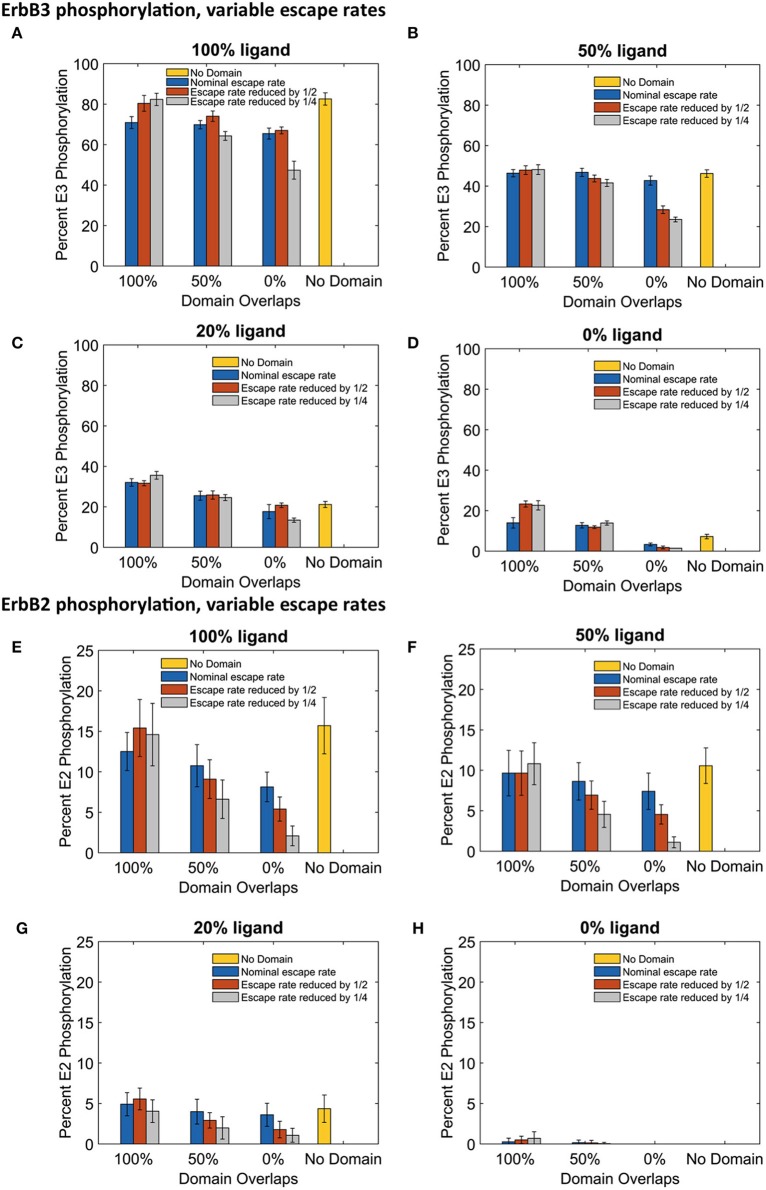
**The effect of changes in domain retention on ErbB3 and ErbB2 phosphorylation across different ligand concentration and domains**. Total receptor phosphorylation across different membrane configurations, ligand concentration and three different escape rates- nominal escape rate (blue bars), escape rate reduced by ½ (orange bars), and escape rate reduced by ½ (gray bars) as well as no domain simulations (yellow bars). **(A)** Total ErbB3 phosphorylation for 100% liganded ErbB3. **(B)** Total ErbB3 phosphorylation for 50% liganded ErbB3. **(C)** Total ErbB3 phosphorylation for 20% liganded ErbB3. **(D)** Total ErbB3 phosphorylation for 0% liganded ErbB3. **(E)** Total ErbB2 phosphorylation for 100% liganded ErbB3. **(F)** Total ErbB2 phosphorylation for 50% liganded ErbB3. **(G)** Total ErbB2 phosphorylation for 20% liganded ErbB3. **(H)** Total ErbB2 phosphorylation for 0% liganded ErbB3. All bars are the averages of 4 runs ± standard deviation.

The significant changes in dimerization with increased domain retention had variable effects on downstream signaling as assessed by steady state phosphorylation levels of ErbB3 and ErbB2 (Figure 6). For ErbB3, phosphorylation levels are relatively stable with increased domain retention (Figures 6A–D). The greatest effect on phosphorylation levels occurred in the case of no domain overlap, where the ErbB3 monomers were more restricted from encounters with ErbB2. In the case of fully-liganded ErbB3, a four-fold reduction in escape rate led to a 28% reduction in phosphorylation (Figure 6A, gray bar for 0% overlap). For lower ligand concentrations, varying domain overlap had a greater effect on phosphorylation than domain retention (Figures 6C,D).

ErbB2 phosphorylation was markedly sensitive to increases in domain retention. Reduced ErbB2 phosphorylation corresponded to decreases in heterodimer formation (Figures 6E–G). Once again, little change was seen with completely overlapping domains. However, increasing domain retention lowered ErbB2 phosphorylation with either partially or non-overlapping domains. Results were striking for simulations run with a four-fold lower escape rate and 100% liganded ErbB3. Here, ErbB2 phosphorylation was reduced by 39% (partially overlapping domains) or 74% (non-overlapping domains).

## Discussion

ErbB2 and ErbB3 are members of the ErbB family of receptor tyrosine kinases that are often co-expressed in cells. Under physiological conditions, neither receptor is active on its own. However, through heterointeractions these receptors activate two key pro-survival pathways. ErbB3 primarily activates the PI3K/Akt pathway and ErbB2 favors the MAP kinase pathway (Yarden and Sliwkowski, 2001). Activation of the ErbB2/ErbB3 signaling unit via overexpression of the receptors, gain-of-function oncogenic mutations, or autocrine release of the ErbB3 ligand, heregulin, have been identified in many types of cancer (Holbro et al., 2003; Wolf-Yadlin et al., 2006; Sheng et al., 2010; Jaiswal et al., 2013; Capparelli et al., 2015). Given the potency of this interaction, normal cells must maintain tight control over ErbB2/ErbB3 interactions. In the absence of ligand, dimerization is limited by the constant fluxing of the ErbB3 extracellular domain from a tethered, inactive conformation to an upright, active conformation with the active conformation stabilized by ligand binding (Dawson et al., 2007). Another way to control ErbB2/ErbB3 interactions may be through dynamic reorganization of membrane domains. Sequestration of ErbB2 and ErbB3 in separate domains could prevent spurious signaling in the absence of ligand, while reorganization into overlapping domains upon ligand binding could encourage the formation of signaling clusters (Vámosi et al., 2006). Evidence for reorganization can be seen in electron microscopy studies of SKBR3 breast cancer cell membranes. ErbB2 and ErbB3 are dispersed in the absence of ligand, but in the presence of ligand, ErbB3 forms large clusters with areas of co-localized ErbB2 and ErbB3 (Yang et al., 2007). It has also been shown that ErbB2 clusters within lipid rafts and that disruption of these rafts reduces both ErbB2 clustering and the association of ErbB2 and ErbB3 (Nagy et al., 2002). The remodeling of domains during active signaling has not yet been explored by simulation, in part due to difficulties in accurately measuring the dynamics of these changes. Here, we have examined how domain remodeling, represented in our model by varying domain overlap and domain retention, will effect heterodimer formation and signaling.

Our spatial stochastic model of ErbB2/ErbB3 interactions provides a useful system in which to explore how changes in domain configuration might affect receptor activation. We began with a model parameterized based on single particle tracking data acquired under low (nanomolar) ligand conditions. We then explored how changes in domain characteristics, as well as ligand occupancy, influences dimerization and phosphorylation in this system. The sensitivity of the model to these parameters illustrates that variations in domain characteristics amongst different cell and tissue types are likely unappreciated modulators of signaling by these (and other) receptors.

Previous spatial stochastic models have shed insight on the effect of domains on signaling (Hsieh et al., 2008; Costa et al., 2009, 2011; Chaudhuri et al., 2011; Kalay et al., 2012). Kalay et al. evaluated movement of tracer molecules within lattice-based domains and found that confinement increased reaction rates (Kalay et al., 2012). Addressing ErbB receptor family interactions with rectangular subdomains, Hsieh et al found that domains created local densities that favored EGFR interactions on the membrane surfaces (Hsieh et al., 2008). Our model increases the complexity by introducing two interacting receptor types with unique behaviors and overlapping, experimentally-defined domains. Thus, the model provides a mechanistic understanding of the interplay between domain overlaps and domain retention on the complex interactions of ErbB2 and ErbB3. The model relies on previously described characteristics of these receptors. For example, ErbB2 homodimers are not favored due to evidence for electrostatic repulsion (Garrett et al., 2003); this translates in the model to a low probability for ErbB2 homointeractions. In addition, ErbB3 has very low kinase activity unless activated by ErbB2 (Steinkamp et al., 2014). Thus, in cells where these are the two predominant ErbB species, they are predicted to be mutually dependent on each other for activation. It follows that differential preference of the two species for unique confinement zones or membrane domains should have a strong influence.

Accordingly, we found that phosphorylation of the two ErbB species was differentially affected by domain overlap. This was particularly evident in the case of 100% liganded ErbB3, where ErbB2 phosphorylation dropped by 50% between completely overlapping to non-overlapping domains (Figure 4D). At these physiological receptor levels, ErbB2 homo-encounters are largely unproductive due to the low on-rate. Simulations with more domain overlap had a larger number of heterodimer interactions than those with partial or no domain overlap. This was most notable when all ErbB3 were occupied with ligand (Figure 4). ErbB3 relies heavily on heterodimerization for activation. However, once ErbB3 receptors are activated by ErbB2, they can go on to homodimerize and activate other ErbB3 receptors. Therefore, steady state ErbB3 phosphorylation was less dependent on domain overlap.

It should be noted that the amount of hetero- and homodimers and phosphorylation levels were nearly the same between no domain spatial stochastic simulations and 100% domain overlapping conditions. This finding differs from our previous work with EGFR which showed that domains greatly improved phosphorylation of EGFR receptors, indicating that the introduction of multiple receptor types to these simulations further complicates outcome (Pryor et al., 2013). True domain overlaps are likely to fall somewhere between non-overlapping and completely overlapping configurations, indicating the need for spatial simulations that take this into account. Ligand binding to ErbB3 in SKBR3 breast cancer cell membranes leads to formation of large ErbB3 clusters with modest levels of co-localized ErbB2; this indicates that domain reorganization can occur during signaling (Yang et al., 2007). The remodeling of domains during active signaling has not yet been explored by simulation, in part due to difficulties in accurately measuring the dynamics of these changes.

SPT has revealed a range of non-brownian motion for proteins on the membrane plane. Anomalous diffusion is a term often used to explain the characteristic restricted movements of proteins that “hop” between membrane domains. There are also reports of specific membrane proteins that undergo directed (motor-driven) motion (Kusumi and Sako, 1996; Saxton and Jacobson, 1997; Schütz et al., 1997; Kusumi et al., 2005). These different modes of motion can have a profound impact on reaction kinetics on the membrane surface by perturbing reaction rates (Saxton and Jacobson, 1997; Melo and Martins, 2006). Thus, it is important to continue evaluating factors, such as diffusion coefficients, corral sizes and escape probability of proteins from their confined domains (Saxton and Jacobson, 1997), that are expected to impact signal initiation and propagation. In this work, we used a simulation approach to study the effect of escape probabilities on the reaction kinetics of the ErbB2/3 signaling pathway. We show that membrane segregation can influence signaling in non-intuitive ways that are linked to the individual characteristics of receptors. Given the technical challenges associated with measuring the dynamics of domain confinement, extent of mixing and escape rates in live cell membranes, simulation offers a powerful tool to explore these variables.

## Author contributions

Conception and design: ÁH, JE. Development of computation framework: RK, ÁH. Acquisition and interpretation of data: RK, ÁH, MS, BW, JE. Writing, review, and/or revision of the manuscript: BW, RK, MS, ÁH, JE. Administrative support and study supervision: BW.

## Funding

This study was supported by National Institutes of Health Grant P50GM085273 (BW), R01GM104973 (JE and ÁH), and R01HG006876 (JE).

### Conflict of interest statement

The authors declare that the research was conducted in the absence of any commercial or financial relationships that could be construed as a potential conflict of interest.
